# Calpain system protein expression in carcinomas of the pancreas, bile duct and ampulla

**DOI:** 10.1186/1471-2407-12-511

**Published:** 2012-11-09

**Authors:** Sarah J Storr, Abed M Zaitoun, Arvind Arora, Lindy G Durrant, Dileep N Lobo, Srinivasan Madhusudan, Stewart G Martin

**Affiliations:** 1 Academic Oncology, University of Nottingham, School of Molecular Medical Sciences, Nottingham University Hospitals NHS Trust, City Hospital Campus, Nottingham, NG5 1PB, UK; 2 Department of Cellular Pathology, Nottingham University Hospitals NHS Trust, Queens Medical Campus, Nottingham, NG7 2UH, UK; 3 Division of Gastrointestinal Surgery, Nottingham Digestive Diseases Centre NIHR Biomedical Research Unit, Nottingham University Hospitals, Nottingham, NG7 2UH, UK; 4 Academic Oncology, University of Nottingham, School of Molecular Medical Sciences, Nottingham University Hospitals NHS Trust, City Hospital Campus, Nottingham, NG5 1PB, UK

**Keywords:** Calpain, Calpastatin, Pancreas, Ampulla, Bile duct, Cancer

## Abstract

**Background:**

Pancreatic cancer, including cancer of the ampulla of Vater and bile duct, is very aggressive and has a poor five year survival rate; improved methods of patient stratification are required.

**Methods:**

We assessed the expression of calpain-1, calpain-2 and calpastatin in two patient cohorts using immunohistochemistry on tissue microarrays. The first cohort was composed of 68 pancreatic adenocarcinomas and the second cohort was composed of 120 cancers of the bile duct and ampulla.

**Results:**

In bile duct and ampullary carcinomas an association was observed between cytoplasmic calpastatin expression and patient age (*P *= 0.036), and between nuclear calpastatin expression and increased tumour stage (*P *= 0.026) and the presence of vascular invasion (*P *= 0.043). In pancreatic cancer, high calpain-2 expression was significantly associated with improved overall survival (*P *= 0.036), which remained significant in multivariate Cox-regression analysis (hazard ratio = 0.342; 95% confidence interva l = 0.157-0.741; *P *= 0.007). In cancers of the bile duct and ampulla, low cytoplasmic expression of calpastatin was significantly associated with poor overall survival (*P *= 0.012), which remained significant in multivariate Cox-regression analysis (hazard ratio = 0.595; 95% confidence interval = 0.365-0.968; *P *= 0.037).

**Conclusion:**

The results suggest that calpain-2 and calpastatin expression is important in pancreatic cancers, influencing disease progression. The findings of this study warrant a larger follow-up study.

## Background

Pancreatic cancer is an aggressive disease with a poor prognosis, with a five year survival rate of 6% in the United States [1]. Although advances in surgery and adjuvant chemotherapy have improved survival rates most patients present with advanced inoperable disease, with poor clinical outcome despite chemotherapy. Chemotherapy in advanced biliary tract cancer improves survival, but the overall prognosis remains poor [2]. The expression of a number of proteins has been shown to be associated with poor survival of patients with pancreatic cancer, including expression of epidermal growth factor receptor (EGFR), insulin-like growth factor-1 receptor (IGF-1R) [3], heat shock protein-27 (HSP27) [4] and VEGF [5]. The role for adjuvant chemotherapy remains unclear in cholangiocarcinomas and ampullary tumours; hence there is an urgent need to develop biomarkers to allow personalised treatments for patients with pancreaticobiliary tumours [6,7]. The calpain system is a family of cysteine proteases, with micro (μ)-calpain and milli (m)-calpain being the most widely studied [8]. Both μ-calpain and m-calpain are heterodimers, each sharing a 28kDa regulatory subunit (*CAPNS1*) and having individual 80kDa catalytic subunit (calpain-1 (*CAPN1*) and calpain-2 (*CAPN2*) respectively). The archetypical family members, μ-calpain and m-calpain, were named on the basis of the concentration of calcium ions required for activation *in-vitro*[8]. There are several mechanisms which can promote calpain activity by reducing the concentration of calcium ions required for activation; these include autolysis of the catalytic subunit, interaction with phospholipids, and phosphorylation [9-11]. Calpastatin (*CAST*) is the ubiquitously expressed inhibitor of μ-calpain and m-calpain, which requires calcium-induced structural changes to μ-calpain and m-calpain for its inhibitory action [12,13].

Many of the precise physiological functions of the calpain family of enzymes remain to be elucidated. In experimental models calpain has been shown to influence cell motility and apoptosis, and as such is implicated in tumour progression and the response of tumour cells to various treatment modalities, including chemotherapy and targeted therapies [14]. Altered expression of the catalytic subunits of μ-calpain and m-calpain, and calpastatin has been described in a number of tumour types including breast cancer [15,16]. The expression of μ-calpain, m-calpain and calpastatin has not been previously examined in pancreatic, ampullary or bile duct cancers, however, a single nucleotide polymorphism of calpain-10 (*CAPN10*) has been associated with an increased risk of developing pancreatic cancer in smokers [17]. In normal pancreatic tissue calpain has been implicated in a number of functions including calcium induced insulin secretion and β-cell spreading [18]. Furthermore, an altered balance between calpastatin and calpain has been implicated in acute pancreatitis in rats [19]. The aims of the current study were to investigate the expression levels of calpastatin, and of the catalytic subunits of μ-calpain and m-calpain in tumours from pancreatic, bile duct and ampullary cancer patients. Furthermore we aimed to determine the importance of expression in terms of associations with clinicopathological variables and clinical outcome.

## Methods

### Clinical samples

Investigation of calpain-1, calpain-2 and calpastatin was conducted using a tissue microarray with tissue collected from patients treated at Nottingham University Hospitals between 1993 and 2010. This study has ethical approval from the Nottingham Research Ethics Committee. This study was conducted according to REMARK criteria [20]. 68 patients with pancreatic adenocarcinoma who underwent surgical resection were included in the study. 65% of patients were male (44/68) and the age of the patients ranged from 35 years to 81 years with a median age of 66. The clinicopathological criteria of the cohort are shown in Table 1. 120 patients with bile duct and ampullary tumours were also included in this study. 54.2% of patients were male (65/120) and the age of the patients ranged from 38 years to 76 years with a median age of 64. The clinicopathological criteria of the cohort are shown in Table 2. For both patient cohorts survival was calculated from the date of surgery to the date of death, or from the date of surgery to the last date known to be alive for those patients censored. The median survival time was 22.5 months for pancreatic adenocarcinomas and 19.6 months for bile duct and ampullary tumours. In the pancreatic cohort 46.9% (23/49) of patients received adjuvant chemotherapy (data was not available for 19 patients), of the 23 patients that did receive adjuvant chemotherapy 78.3% (18/23) received 5FU/folinic acid and 21.7% (5/23) received gemcitabine chemotherapy. In the bile duct and ampullary cohort 24.6% (17/69) of patients received adjuvant chemotherapy (data was not available for 51 patients), of the 17 patients that did receive adjuvant chemotherapy 76.5% (13/17) received 5FU/folinic acid and 23.5% (4/17) received gemcitabine chemotherapy in the context of clinical trials. The operative intention was curative and patients with suspected involvement of the portal vein following assessment by radiology did not receive surgery. In the pancreatic cohort 82% (56/68) of patients had whipple surgery, 10% (7/68) of patients had a distal pancreatectomy and 7% (5/68) of patients had a total pancreatectomy. In the ampulla and bile duct cohort 98% (110/112) of patients had whipple surgery and 2% (2/112) of patients had a total pancreatectomy. In the pancreatic cohort 44.1% (30/68) of patients had satisfactory margins following surgery (R0) and 55.9% (38/68) of patients had margins that were compromised (R1). In the ampulla and bile duct cohort 64.7% of patients (77/120) had satisfactory margins (R0) and 35.3% (42/120) of patients had compromised margins (R1).

**Table 1:** Associations between calpastatin, calpain-1 and calpain-2 protein expression and various clinicopathological variables in the pancreatic cancer cohort

	**Calpain-1**			**Calpain-2**			**Calpastatin (cytoplasmic)**			**Calpastatin (nuclear)**		
	**Low**	**High**	**p value**	**Low**	**High**	**p value**	**Low**	**High**	**p value**	**Low**	**High**	**p value**
**Age**												
≤ 60 years (n=23)	17 (34.7)	4 (30.8)	1.000*	3 (18.8)	18 (37.5)	0.225*	16 (32.0)	4 (33.3)	1.000*	15 (33.3)	5 (29.4)	1.000*
> 60 years (n=45)	32 (65.3)	9 (69.2)		13 (81.3)	30 (62.5)		34 (68.0)	8 (66.7)		30 (66.7)	12 (70.6)	
**Tumour size**												
≤ 2cm (n=17)	16 (33.3)	1 (7.7)	0.088*	5 (31.3)	12 (25.5)	0.747*	13 (26.5)	3 (25.0)	1.000*	10 (22.7)	6 (35.3)	0.317
> 2cm (n=50)	32 (66.7)	12 (92.3)		11 (68.8)	35 (74.5)		36 (73.5)	9 (75.0)		34 (77.3)	11 (64.7)	
**T stage**												
2 (n=12)	6 (12.2)	3 (23.1)	0.335	2 (12.5)	9 (18.8)	0.576	8 (16.0)	3 (25.0)	0.380	8 (17.8)	3 (17.6)	0.766
3 (n=54)	42 (85.7)	9 (69.2)		14 (87.5)	37 (77.1)		41 (82.0)	8 (66.7)		36 (80.0)	13 (76.5)	
4 (n=2)	1 (2.0)	1 (7.7)		0 (0.0)	2 (4.2)		1 (2.0)	1 (8.3)		1 (2.2)	1 (5.9)	
**N stage**												
Negative (n=22)	17 (35.4)	4 (33.3)	1.000*	3 (18.8)	18 (39.1)	0.220*	15 (30.6)	5 (45.5)	0.481*	14 (31.8)	6 (37.5)	0.760*
Positive (n=44)	31 (64.6)	8 (66.7)		13 (81.3)	28 (60.9)		34 (69.4)	6 (54.5)		30 (68.2)	10 (62.5)	
**Vascular invasion**												
absent (n=24)	19 (38.8)	4 (30.8)	0.751*	3 (18.8)	20 (41.7)	0.136*	19 (38.0)	4 (33.3)	1.000*	15 (33.3)	8 (47.1)	0.318
present (n=44)	30 (61.2)	9 (69.2)		13 (81.3)	28 (58.3)		31 (62.0)	8 (66.7)		30 (66.7)	9 (52.9)	
**Perineural invasion**												
absent (n=14)	8 (16.3)	5 (38.5)	0.122*	1 (6.3)	12 (25.0)	0.157*	11 (22.0)	2 (16.7)	1.000*	10 (22.2)	3 (17.6)	1.000*
present (n=54)	41 (83.7)	8 (61.58)		15 (93.8)	36 (75.0)		39 (78.0)	10 (83.3)		35 (77.8)	14 (82.4)	

The frequency of observed clinicopathological variables is noted next to the variable subgroup; one case did not have available data for tumour size. The *P* values are resultant from Pearson Chi Square test of association (χ^2^) or Fisher’s Exact test in a 2×2 table if a cell count was less than 5 (indicated by *). Significant *P* values are indicated by bold font.

**Table 2:** Associations between calpastatin, calpain-1 and calpain-2 protein expression and various clinicopathological variables in the bile duct and ampullary cancer cohort

**Variable**	**Calpain-1**			**Calpain-2**			**Calpastatin (cytoplasmic)**			**Calpastatin (nuclear)**		
	**Low**	**High**	**p value**	**Low**	**High**	**p value**	**Low**	**High**	**p value**	**Low**	**High**	**p value**
**Age**												
≤ 60 years (n=40)	12 (30.0)	27 (38.6)	0.366	14 (36.8)	22 (34.9)	0.845	16 (50.0)	22 (28.9)	**0.036**	25 (35.7)	12 (32.4)	0.734
> 60 years (n=78)	28 (70.0)	43 (61.4)		24 (63.2)	41 (65.1)		16 (50.0)	54 (71.1)		45 (64.3)	25 (67.6)	
**Tumour size**												
≤ 2cm (n=48)	15 (37.5)	29 (40.8)	0.729	18 (46.2)	21 (33.3)	0.195	17 (51.5)	27 (35.5)	0.118	30 (42.3)	13 (35.1)	0.473
> 2cm (n=70)	25 (62.5)	42 (59.2)		21 (53.8)	42 (66.7)		16 (48.5)	49 (64.5)		41 (57.7)	24 (64.9)	
**T stage**												
1 (n=3)	0 (0.0)	3 (4.2)	0.262	0 (0.0)	3 (4.7)	0.137	1 (3.0)	2 (2.6)	0.918	0 (0.0)	3 (8.1)	**0.026**
2 (n=27)	11 (26.8)	13 (18.3)		7 (17.9)	15 (23.4)		8 (24.2)	17 (22.1)		20 (27.8)	5 (22.9)	
3 (n=86)	28 (68.3)	54 (76.1)		30 (76.9)	46 (71.9)		23 (69.7)	57 (74.0)		50 (69.4)	29 (78.4)	
4 (n=3)	2 (4.9)	1 (1.4)		2 (5.1)	0 (0.0)		1 (3.0)	1 (1.8)		2 (2.8)	0 (0.0)	
**N stage**												
Negative (n=44)	14 (35.9)	28 (41.2)	0.590	14 (37.8)	24 (39.3)	0.882	11 (35.5)	30 (40.5)	0.628	26 (38.2)	15 (41.7)	0.733
Positive (n=69)	25 (64.1)	40 (58.8)		23 (62.2)	37 (60.7)		20 (64.5)	44 (59.5)		42 (61.8)	21 (58.3)	
**Vascular invasion**												
absent (n=52)	18 (43.9)	30 (42.9)	0.915	18 (47.4)	26 (40.6)	0.506	13 (39.4)	34 (44.7)	0.605	36 (50.0)	11 (29.7)	**0.043**
present (n=66)	23 (56.1)	40 (57.1)		20 (52.6)	38 (59.4)		20 (60.6)	42 (55.3)		36 (50.0)	26 (70.3)	
**Perineural invasion**												
absent (n=51)	17 (41.5)	32 (45.1)	0.711	16 (41.0)	30 (46.9)	0.562	15 (45.5)	34 (44.2)	0.900	36 (50.0)	12 (32.4)	0.080
present (n=68)	24 (58.5)	39 (54.9)		23 (59.0)	34 (53.1)		18 (54.5)	43 (55.8)		36 (50.0)	25 (67.6)	
**Tumour site**												
Proximal bile duct (n=5)	3 (7.3)	1 (1.4)	0.251	1 (2.6)	3 (4.7)	0.418	3 (9.1)	1 (1.3)	0.074	4 (5.6)	0 (0.0)	0.288
Distal bile duct (n=65)	22 (53.7)	38 (53.5)		24 (61.5)	31 (48.4)		19 (57.6)	39 (50.6)		39 (54.2)	19 (51.4)	
Ampullary (n=50)	16 (39.0)	32 (45.1)		14 (35.9)	30 (46.9)		11 (33.3)	37 (48.1)		29 (40.3)	18 (48.6)	

The frequency of observed clinicopathological variables is noted next to the variable subgroup; in some instances data was not available, 2 cases for patient age, 2 cases for tumour size: 1 case for tumour stage, 7 cases for N stage, 2 cases for vascular invasion, and 1 case for perineural invasion. The *P* values are resultant from Pearson Chi Square test of association (χ^2^) or Fisher’s Exact test in a 2×2 table if a cell count was less than 5 (indicated by *). Significant *P* values are indicated by bold font.

### Tissue microarray and immunohistochemistry

The tissue microarray (TMA) was prepared using triplicate 0.6mm tissue cores of tumour, identified by a specialist pathologist, placed into a single recipient paraffin block. 4μm sections of the TMA were mounted on poly-L-lysine coated slides. Immunohistochemistry was performed on the TMA slides which were initially deparaffinised in xylene followed by rehydration in ethanol. Antigen retrieval was performed in 0.01molL^-1^ sodium citrate buffer (pH6) in a microwave; 450W for 10 minutes. Endogenous peroxidase activity was blocked over 10 minutes in 0.01% hydrogen peroxide in methanol. Primary antibodies; mouse anti-calpastatin (1:15,000), mouse anti-calpain-1 (1:2500) and rabbit anti-calpain-2 (1:2500) (all Chemicon, Massachusetts, USA, clones PI-11, P-6 and rabbit polyclonal AB1625 respectively with specificity confirmed by Western blotting) were diluted in blocking serum and applied to the tissue for one hour at room temperature. Staining was achieved using the Vectastain Elite ABC kit (universal), containing blocking serum, biotinylated secondary antibody and ABC reagent (Vector Laboratories, Peterborough, UK). Immunohistochemical reactions were developed with 3,3’ diaminobenzidine as the chromogenic peroxidase substrate (Dako, Glostrup, Denmark). Sections were then counterstained with Gills formula Haematoxylin (Vector Laboratories), dehydrated and fixed in xylene prior to mounting with DPX. Breast tumour composite sections which comprised of 6 stage 1 breast tumours of grade 1 to 3 were included as positive and negative controls with each run, with the negative control having primary antibody substituted for PBS. All cores were assessed semi-quantitatively using an immunohistochemical H-score using a Nikon Eclipse E600 at 200x magnification. Staining intensity was assessed as; none (0), weak (1), medium (2) and strong (3) over the percentage area of each staining intensity. H scores were calculated by multiplying the percentage area by the intensity grade (H score range 0–300). Each core was assessed by two individuals, including one pathologist and a consensus agreed. An average H-score was generated by taking the mean H-score of the three cores available for each patient.

### Statistical analysis

The relationship between categorised protein expression and clinicopathological variables was assessed using Pearson Chi Square (χ^2^) test of association. Survival curves were plotted according to the Kaplan-Meier method and significance determined using the log-rank test. Multivariate survival analysis was performed by Cox Proportional Hazards regression model. All differences were deemed statistically significant at the level of *P*<0.05. Statistical analysis was performed using SPSS 19.0 software (IBM Corporation, NY, USA). Stratification cut-points were determined using X-Tile software (Yale School of Medicine, CT, USA) and were determined prior to statistical analyses [21].

## Results

### Staining location and frequency

**Figure 1:**
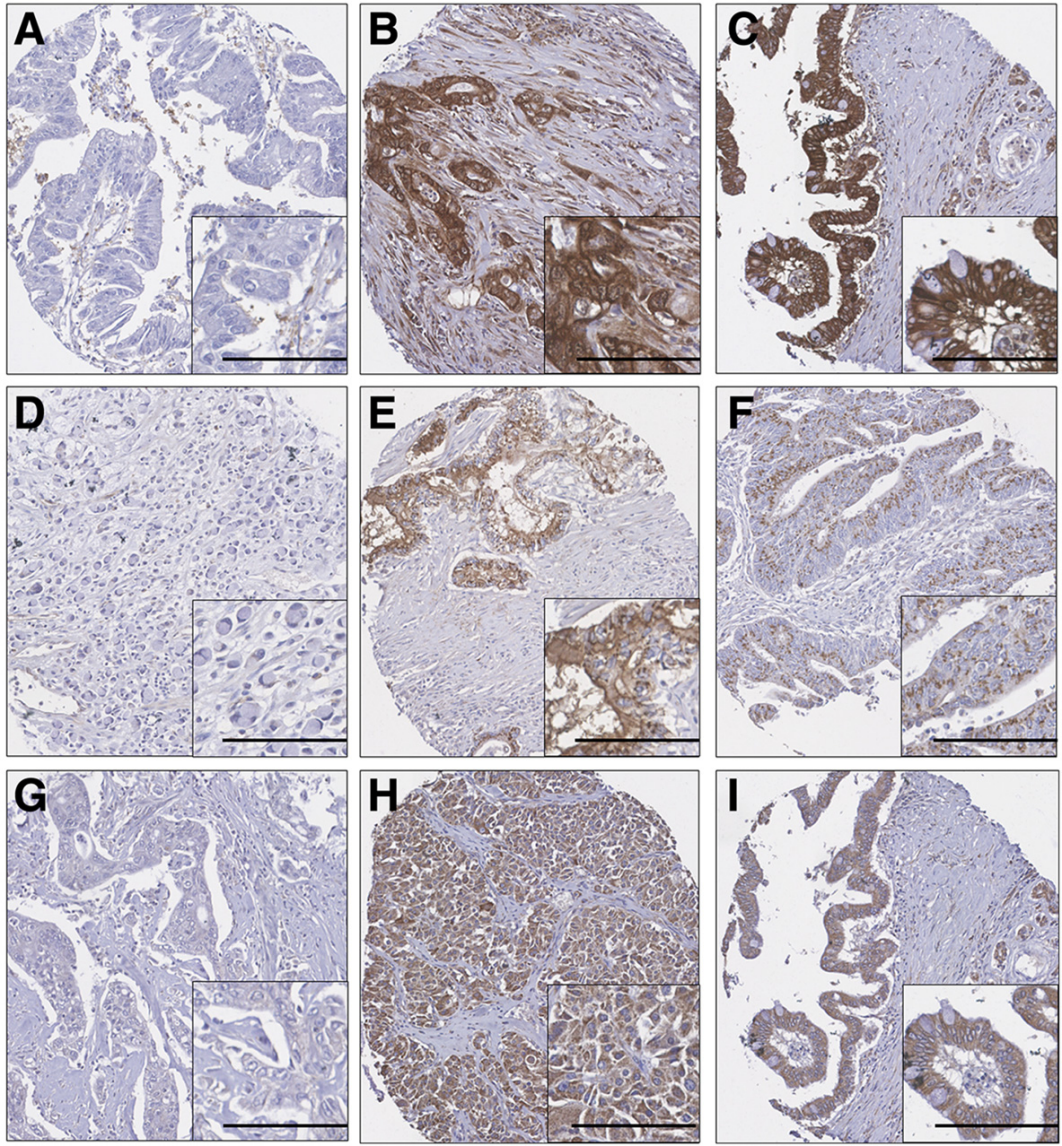
**Representative photomicrographs of protein expression.A**-**C**: calpastatin staining in pancreatic and distal cholangiocarcinoma; A: absence of immunoreactivity in papillary pancreatic adenocarcinoma; **B**: strong cytoplasmic immunoreactivity in moderately differentiated cholangiocarcinoma; **C**: staining in mucinous secreting adenocarcinoma. **D**-**F**: calpain-1 staining in pancreatic carcinoma; **D**: absence of immunoreactivity in poorly differentiated adenocarcinoma; **E**: moderate cytoplasmic and nuclear staining in moderately differentiated adenocarcinoma; **F**: weak dot cytoplasmic immunoreactivity in pancreatic adenocarcinoma. **G**-**I**: calpain-2 staining in pancreatic carcinoma; **G**: absence of immunoreactivity in moderately differentiated adenocarcinoma; **H**: moderate cytoplasmic and nuclear activity in poorly differentiated adenocarcinoma; **I**: staining in mucinous secreting adenocarcinoma. Photomicrographs are at 10x magnification with 20x magnification inset box where scale bar shows 100μm.

Calpain-1 and calpain-2 demonstrated cytoplasmic staining with some granularity and heterogeneity between adjacent tumour cells, varying from weak to intense staining with a few instances of calpain-2 nuclear staining (Figure 1). Calpastatin stained both the cytoplasm and nucleus of tumour cells. In the pancreatic adenocarcinoma cohort calpain-1 had a median H-score of 140 and ranged from 0 to 280; calpain-2 had a median H-score of 118 and ranged from 10 to 293; cytoplasmic calpastatin expression had a median H-score of 188 and ranged from 0 to 300 and nuclear calpastatin expression had a median H-score of 54 and ranged from 0 to 300. The X-tile cut point for calpain-1 was 200, calpain-2 was 80, cytoplasmic calpastatin was 285 and nuclear calpastatin was 110; with 21.0% (13/62), 75.0% (48/64), 19.4% (12/62), and 27.4% (17/62) respectively having high protein expression. In the bile duct and ampullary carcinoma cohort calpain-1 had a median H-score of 100 and ranged from 0 to 270; calpain-2 had a median H-score of 50 and ranged from 0 to 200; cytoplasmic calpastatin expression had a median H-score of 137 and ranged from 0 to 300 and nuclear calpastatin expression had a median H-score of 20 and ranged from 0 to 210. The X-tile cut point for calpain-1 was 70, calpain-2 was 50, cytoplasmic calpastatin was 103 and nuclear calpastatin was 40; with 63.4% (71/112), 62.1% (64/103), 70.0% (77/110), and 33.9% (37/109) respectively having high protein expression. The correlation between expression of the proteins with each other was assessed using the Spearman rank correlation coefficient. In the pancreatic cohort calpain-1 expression had a statistically significant correlation, but of marginal biological importance, with calpain-2 expression (r=0.281, *P*=0.026), cytoplasmic calpastatin expression (r=0.327, *P*=0.010), and nuclear calpastatin expression (r=0.346, *P*=0.006). No correlation was observed between calpain-2 expression and cytoplasmic or nuclear calpastatin expression. Cytoplasmic expression of calpastatin was strongly correlated with nuclear calpastatin expression (r=0.824, *P*<0.001). In the bile duct and ampullary carcinomas calpain-1 expression had a statistically significant correlation with cytoplasmic calpastatin expression (r = 0.425, *P *< 0.001) and nuclear calpastatin expression (r = 0.295, *P *= 0.002), but not with calpain-2 expression. Calpain-2 expression was correlated with cytoplasmic calpastatin expression (r = 0.250, *P *= 0.009) but not nuclear calpastatin expression. Cytoplasmic expression of calpastatin was strongly correlated with nuclear calpastatin expression (r = 0.665, *P *< 0.001).

### Clinicopathological criteria

The expression of calpain-1, calpain-2 and calpastatin cytoplasmic and nuclear expression was tested to determine associations with clinicopathological criteria in the pancreatic and the grouped bile duct and ampullary cancers. No associations were observed between marker expression and pancreatic adenocarcinomas (Table 1). In the bile duct and ampullary carcinomas an association was observed between high cytoplasmic calpastatin expression and patients aged above 60 years (χ^2^=4.376, d.f.=1, *P*=0.036). In addition an association between high nuclear calpastatin expression and increased tumour stage (χ^2^=9.303, d.f.=3, *P*=0.026) and the presence of vascular invasion (χ^2^=4.093, d.f.=1, *P*=0.043) (Table 2).

### Relationship with clinical outcome

**Figure 2:**
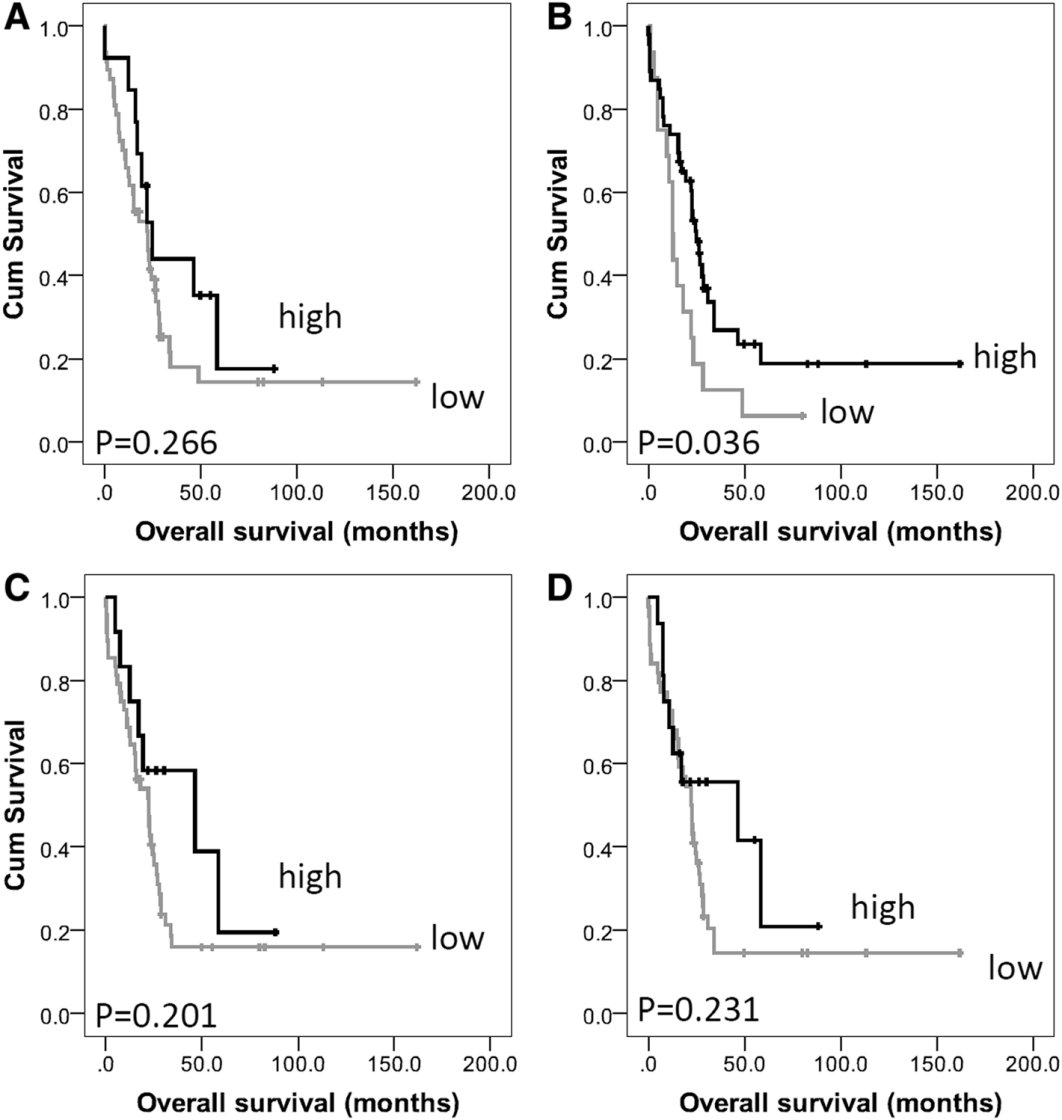
Kaplan-Meier analysis of progression-free survival showing the impact of calpain-1 (panel A), calpain-2 (panel B), cytoplasmic calpastatin (panel C) and nuclear calpastatin (panel D) expression in the pancreatic cancer cohort with significance determined using the log rank test.

**Figure 3:**
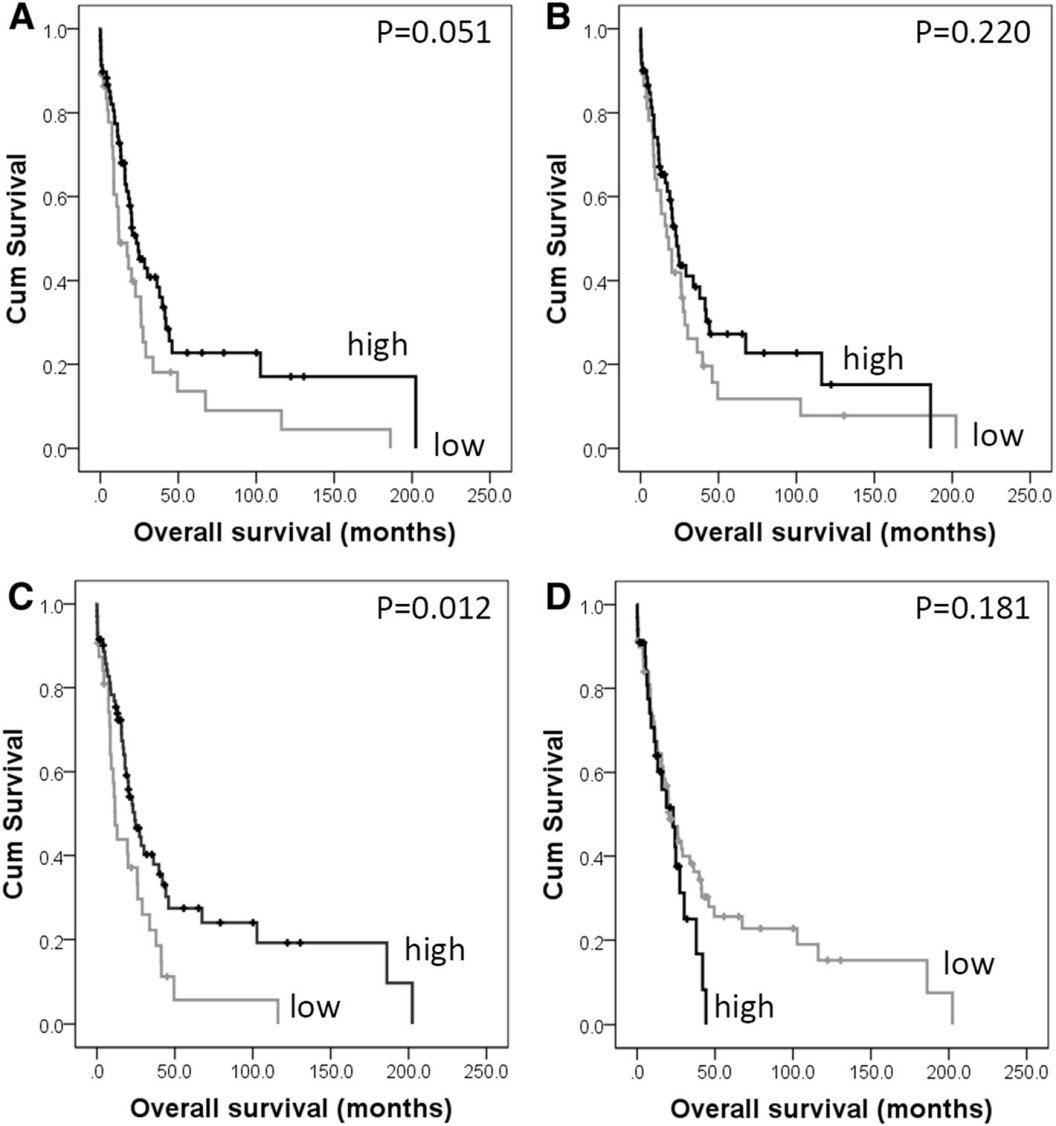
Kaplan-Meier analysis of progression-free survival showing the impact of calpain-1 (panel A), calpain-2 (panel B), cytoplasmic calpastatin (panel C) and nuclear calpastatin (panel D) expression in the bile duct and ampullary cancer cohort with significance determined using the log rank test.

In pancreatic cancer calpain-2 was significantly associated with overall survival (*P *= 0.036) (Figure 2, panel A), which remained significant in multivariate Cox-regression analysis (Hazard Ratio (HR) = 0.342; 95% Confidence Interval (95% CI) = 0.157-0.741; *P *= 0.007) (Table 3 panel A). In the multivariate Cox-regression the potential confounding factors of patient sex (*P *= 0.119) and age (*P *= 0.683), tumour size (*P *= 0.086), grade (*P *= 0.824), stage (*P *= 0.677), lymph node status (*P *= 0.127), perineural and vascular invasion (*P *= 0.152 and *P *= 0.449) were included; none of these parameters were significantly associated with survival. In cancers of the bile duct and ampulla, low cytoplasmic expression of calpastatin was significantly associated with poor overall survival (*P *= 0.012) (Figure 3, panel C), which remained significant in multivariate Cox-regression analysis (HR = 0.595; 95% CI = 0.365-0.968; *P *= 0.037) (Table 3 panel B). In the multivariate Cox-regression the potential confounding factors of tumour stage, perineural invasion and tumour grade were included (with individual Kaplan-Meier statistics of *P *= 0.037, *P *=0.041 and *P *= 0.048 respectively). When the ampullary and bile duct cohort was separated to cancers of the ampulla and cancers of the proximal and distal bile duct, low calpastatin expression was associated with survival in the ampullary cancers only (*P *= 0.043). Resection margin was available for both the pancreatic and ampulla and bile duct cohorts, however was not included in multivariate analysis as it was not significantly associated with survival (*P *= 0.912 and *P *= 0.446 respectively); similar results were observed for adjuvant chemotherapy, with adjuvant chemotherapy not associated with survival in the pancreatic cohort (*P*=0.052), or the ampulla and bile duct cohort (*P*=0.430).

**Table 3:** Cox proportional hazards analysis for overall survival in the pancreatic cancer cohort (A) and the bile duct and ampullary cancer cohort (B) for calpain-2 and calpastatin expression respectively

**A**					
		**p value**	**Exp(B)**	**95.0% CI for Exp(B)**	
				**Lower**	**Upper**
	Calpain 2 expression	**0.007**	0.342	0.157	0.741
	Sex	0.042	2.136	1.029	4.433
	T stage	0.672	1.242	0.456	3.380
	Node status	0.224	1.642	0.738	3.653
	Vascular invasion	0.683	0.868	0.439	1.715
	Perineural invasion	0.946	1.030	0.434	2.442
	Grade	0.211	1.481	0.801	2.741
	Tumour size	0.017	2.929	1.209	7.098
	Patient age	0.332	0.709	0.353	1.421
**B**					
		**p value**	**Exp(B)**	**95.0% CI for Exp(B)**	
				**Lower**	**Upper**
	Cytoplasmic calpastatin	**0.037**	0.595	0.365	0.968
	T stage	0.083	1.525	0.946	2.459
	Perineural invasion	0.388	1.251	0.752	2.083
	Grade	0.124	1.422	0.908	2.225

Exp (B) is used to denote hazard ratio, and 95% CI is used to denote 95% confidence interval.

## Discussion

This study examined the expression of calpain-1 (μ-calpain catalytic subunit), calpain-2 (m-calpain subunit) and calpastatin in cancers of the pancreas, bile duct and ampulla using immunohistochemistry. No associations were observed between protein expression of the calpain system and clinicopathological criteria of the pancreatic adenocarcinoma cohort. In the bile duct and ampulla carcinoma cohort a significant association was observed between high cytoplasmic calpastatin expression and patients aged above 60 years and high nuclear calpastatin expression and increased tumour stage and the presence of vascular invasion. An association between low cytoplasmic calpastatin expression and the presence of lymphovascular invasion, encompassing invasion of both lymphatic and blood vessels has been reported in breast cancer [15]. It is interesting to note that the current results show a high frequency of calpastatin expression within the nucleus, that has not previously been observed in other tumour types, such as breast cancer [15]. Perineural aggregation of calpastatin has been reported in brain cells, whereby aggregation serves as an intracellular store of the inhibitor prior to its release into the cytosol [22]. Furthermore there is evidence to suggest that nuclear translocation of calpastatin occurs in experimental models [23,24]. It is unclear as to why this would occur more often in cancers of the pancreas, bile duct and ampulla. The calpain system, including calpastatin, is implicated in tumour progression through its role in multiple cell pathways including cellular migration, apoptosis and cell survival [14]. There is little information in pancreatic, bile duct or ampullary cancers regarding calpain function or activity; however, single nucleotide polymorphisms in calpain-10 (*CAPN10*) have been associated with pancreatic cancer in smokers [17]. In this study low calpain-2 expression was associated with adverse overall survival in the pancreatic cancer cohort (*P*=0.036), which remained significant in multivariate analysis (*P*=0.007). This was perhaps unexpected as previous findings in breast cancer have indicated that high calpain expression is associated with poor survival [16]. It is unclear why low levels of calpain-2 expression are associated with poor survival outcome, although it may be due to numerous factors such as the tumour type, the chemotherapy treatment or lack thereof, or simply the nature of the organ. A further important aspect to note is that this study describes the protein expression of calpain-1 and calpain-2 and not the relative calpain activity levels. Determining the activity of calpain may be possible as part of future studies, using antibodies against calpain specific degradation products; however such reagents require further validation in human malignancies such as cancer [25,26]. In the bile duct and ampullary cancer cohort, low cytoplasmic calpastatin expression was associated with adverse overall survival (*P*=0.012), which remained significant in multivariate analysis (*P*=0.037). In the ampulla and bile duct cohort only tumour stage, grade and perineural invasion was associated with survival and included in the multivariate analysis; this may be due to the earlier presentation of cancers in comparison to pancreatic adenocarcinoma due to the manifestation of clinical symptoms. Low calpain expression would be expected to allow a higher level of calpain activity, however in cases where low expression of calpastatin was observed, low calpain-1 and calpain-2 protein expression was observed. As previously stated this study determined the protein expression level of calpain-1 and calpain-2 and as such the finding that low calpastatin expression is linked with low calpain-1 and calpain-2 expression may suggest that protein expression and activity of the enzymes are somewhat discordant.

In summary, this study demonstrates that low expression of calpain-2 is associated with poor survival in pancreatic cancer and low cytoplasmic calpastatin expression is associated with poor survival in cancers of the bile duct and ampulla. The associations observed between protein expression and adverse survival outcome remained significant in multivariate analysis. Determining expression of the calpain system may be of benefit in patients with pancreatic, bile duct of ampullary cancers. The findings of this study warrant a larger follow-up study with increased numbers of patients.

## Competing interest

The authors declare that they have no competing interest.

## Authors’ contributions

SJS carried out the staining; SJS and AMZ carried out the scoring; AMZ, AA, DNL, SM carried out data collection; SJS, AMZ, MS, SGM participated in data analysis; SJS, AMZ, LGD, DNL, SM, SGM participated in design and coordination of the study. SGM conceived of the study. All authors read and approved the final manuscript.

## Pre-publication history

The pre-publication history for this paper can be accessed here:

http://www.biomedcentral.com/1471-2407/12/511/prepub
